# Characterization of murine macrophages from bone marrow, spleen and peritoneum

**DOI:** 10.1186/1471-2172-14-6

**Published:** 2013-02-05

**Authors:** Changqi Wang, Xiao Yu, Qi Cao, Ya Wang, Guoping Zheng, Thian Kui Tan, Hong Zhao, Ye Zhao, Yiping Wang, David CH Harris

**Affiliations:** 1 Centre for Transplant and Renal Research at Westmead, Sydney, NSW, Australia; 2 Department of Urology, Tongji Hospital, Tongji Medical College, Huazhong University of Science and Technology, Wuhan, PR China; 3 Department of Biochemistry and Molecular Biology, Shanxi Medical University, Shanxi, PR China

**Keywords:** Macrophage, Bone marrow, Spleen, Peritoneum

## Abstract

**Background:**

Macrophages have heterogeneous phenotypes and complex functions within both innate and adaptive immune responses. To date, most experimental studies have been performed on macrophages derived from bone marrow, spleen and peritoneum. However, differences among macrophages from these particular sources remain unclear. In this study, the features of murine macrophages from bone marrow, spleen and peritoneum were compared.

**Results:**

We found that peritoneal macrophages (PMs) appear to be more mature than bone marrow derived macrophages (BMs) and splenic macrophages (SPMs) based on their morphology and surface molecular characteristics. BMs showed the strongest capacity for both proliferation and phagocytosis among the three populations of macrophage. Under resting conditions, SPMs maintained high levels of pro-inflammatory cytokines expression (IL-6, IL-12 and TNF-α), whereas BMs produced high levels of suppressive cytokines (IL-10 and TGF-β). However, SPMs activated with LPS not only maintained higher levels of (IL-6, IL-12 and TNF-α) than BMs or PMs, but also maintained higher levels of IL-10 and TGF-β.

**Conclusions:**

Our results show that BMs, SPMs and PMs are distinct populations with different biological functions, providing clues to guide their further experimental or therapeutic use.

## Background

Macrophages play an essential role in both innate and adaptive immunity [1]. Macrophages are the indispensable part of the host defense system because of their presence in virtually every type of tissue, their capacity to contain the majority of infections in the early phase of their development, and their ability to mount specific immunological responses.

Macrophages are distributed in all tissues and organs after birth. The distribution patterns of macrophages have been shown by labeling the colony-stimulated factor 1 receptor (Csf1r) promoter with green fluorescent protein (GFP) [2] or by specific F4/80 antibody (Ab) staining of macrophages [3]. It has been found that distinctive morphological differences within and among macrophage populations could be attributed to their heterogeneity [4]. The heterogeneity of macrophages may be important for their diverse and flexible participation in immune responses. Therefore, it is important to examine the phenotypic and functional differences amongst macrophages from different origins, such as spleen, bone marrow and peritoneum.

Peritoneal macrophages (PMs) have been widely used as a macrophage source in mice since the 1960s [5,6]. Possibly due to the low organ tension within the peritoneal cavity, PMs are remarkably distinct from macrophages of other tissues [7]. For example, PMs have higher expression of inducible nitric oxide synthase and IL-12 than do splenic macrophages (SPMs) [8].

SPMs were originally located in the cords of Billroth in splenic red pulp and termed red pulp macrophages, which show a high acid phosphatase activity and several detectable macrophage markers, such as F4/80, Mac-1 and MOMA-2 [9-12]. Previous studies have found that SPMs differ significantly from PMs in their requirements for activation [13], and exhibit different levels of CD40L, IL-1 and scavenger receptors [14,15]. It has been reported in a tumor-bearing mouse study, that cytotoxicity was significantly decreased in PMs,while markedly increased in SPMs [16]. However, the differences of SPMs with other resident macrophages have not been fully addressed.

Another source for commonly used macrophages is the bone marrow. The growth of bone marrow macrophages (BM) requires macrophage colony-stimulating factor (M-CSF). In the past, studies of macrophages have had a bias towards macrophages derived from one specific organ. For instance, BMs have been commonly used due to their homogeneity, ability to be transfected, proliferation capacity and longer lifespan. However, the application of BMs in experimental studies also has difficulty due to the instability of their phenotype and functions in vivo [17]. BMs are relatively flexible in their response to modification; for example, their proliferation can be regulated by changing the concentration of growth factor M-CSF [18].

For those reasons, it is important to define differences among macrophages derived from spleen, bone marrow and peritoneal cavity. The aim of this study was to explore differences in morphology, phenotype, proliferation, phagocytosis, antigen presentation and cytokine expression of murine SPMs, BMs and PMs.

## Results

### Morphological difference of SPMs, BMs and PMs

**Figure 1:**
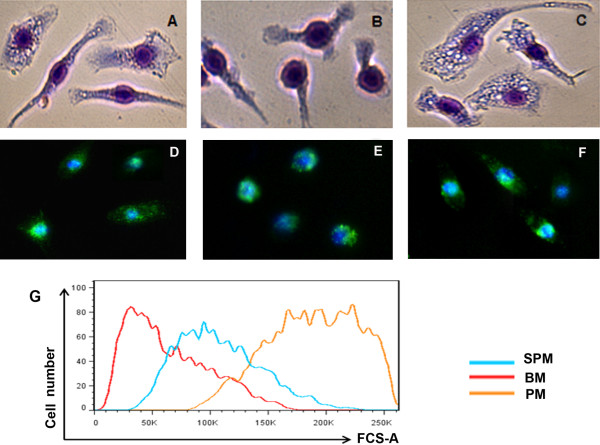
**Morphological characteristics of cultured macrophages derived from spleen (A, D), bone marrow (B, E) and peritoneal cavity (C, F), and their cell size assessment (G). **All cells were cultured in complete RPMI1640 on 6-well plates, and after removal of supernatant, cells were then stained with Giemsa-wright dye **(A, B, C) **and to demonstrate lysosome, anti-LAMP1 **(D, E, F) **(original magnification x400). Cell size was assessed by flow cytometry analysis **(G).**

PMs displayed a larger cell size (Figure 1G) and higher lysosomal content than both SPMs and BMs (Figure 1D, E and F). SPMs had a more elongated spindle shape than PMs and BMs (Figure 1A, B and C), and lower lysosomal content. BMs contained less cytoplasm than PMs or SPMs.

### Phenotype differences of SPMs, PMs and BMs

**Figure 2:**
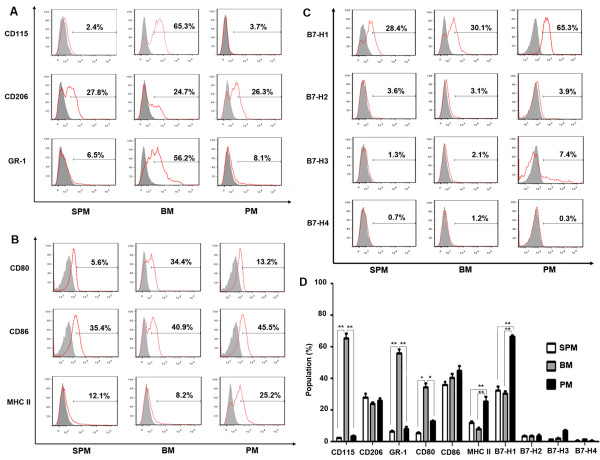
**Expression of surface molecules on resting SPM, BM and PM was determined by flow cytometry. **Red solid lines, staining with **(A) **anti-CD115, anti-CD206, anti-Gr-1, **(B) **anti-CD80, anti-CD86, anti-MHC II, **(C)** anti-B7-H1, anti-B7-H2, anti-B7-H3 and anti-B7-H4; grey filled , staining with the relevant isotype controls. The percentage positivity is shown at the upper right of each histogram. Data are representative of 5 separate experiments of each macrophage preparation. **D**: summary data of surface molecules expression. Data are mean ± SEM. *p < 0.05, **p < 0.01.

The expression of CD115, CD206, GR-1, CD80, CD86, MHCII, B7-H1, B7-H2, B7-H3 and B7-H4 was examined by flow cytometry analysis. CD115 was expressed frequently on BMs (65.4 ± 3.0%), and significantly less on SPMs (2.4 ± 0.4%) and PMs (3.6 ± 0.2%). Similarly, Gr-1 exhibited a much more frequent expression on BMs (56.2 ± 2.3%) than on SPMs (6.6 ± 0.7%) or PMs (8.3 ± 1.1%) (Figure 2A, D).

CD80, CD86 and MHC II are important costimulatory molecules for T cell stimulation. PMs demonstrated high frequent expression of MHC II (25.5 ± 3.2%) and CD86 (45.3 ± 2.7%), whereas, BMs had high expression of CD80 (34.6 ± 2.6%). SPMs showed relatively low expression of CD80 (5.5 ± 0.8%) and CD86 (36.1 ± 1.9%) (Figure 2B, D).

Expression of other costimulatory ligands including B7-H1, B7-H2, B7-H3 and B7-H4 was examined by flow cytometry. The expression of B7-H1 was much more frequent on PMs (66.7 ± 0.8%) than SPMs (32.5 ± 2.5%) or BMs (30.7 ± 1.3%). Low expression level of B7-H2, B7-H3 and B7-H4 was shown for all three macrophage types (Figure 2C, D).

### Proliferative capability of SPMs, BMs and PMs

**Figure 3:**
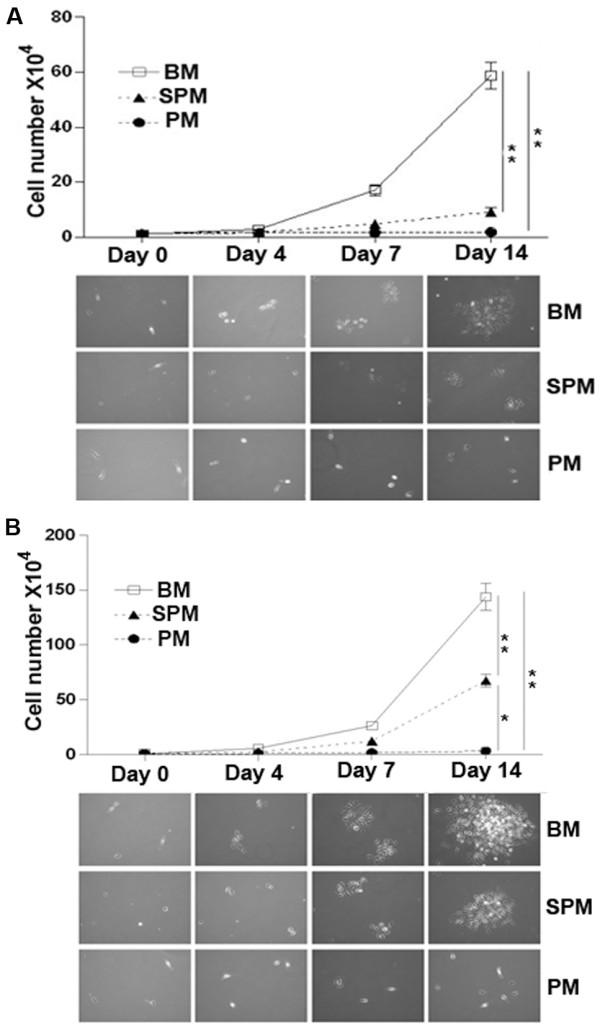
**Macrophage growth rate treated with different M-CSF concentrations. **BM, SPM and PM were cultured with M-CSF in concentrations of 2 ng/ml **(A) **or 10 ng/ml **(B) **for 0, 4, 7 and 14 days. The numbers of macrophages were quantified. Images are representative of 3 separate experiments. Data are mean ± SEM. *p < 0.05, **p < 0.01.

The proliferative capability of SPMs, BMs and PMs was assessed. Under culture with 2 ng/ml M-CSF (Figure 3A), BMs showed a much stronger proliferative capability than SPMs and PMs. BM numbers increased from day 4, and continued until to day14 when there was a 60 fold increase over baseline. However, SPMs showed less proliferation with only a 7 fold increase. In contrast, there was no proliferation in PMs during the 14 day culture (Figure 3B).

In response to 10 ng/ml of M-CSF (Figure 3B), the proliferation of the three macrophage populations showed similar patterns to those with 2 ng/ml M-CSF. The proliferation of BMs and SPMs was much greater than that in low concentration M-CSF (Figure 3B). However, an increase of M-CSF concentration up to 10 ng/ml did not enhance proliferation capability of PMs.

### Capacity of phagocytosis

**Figure 4:**
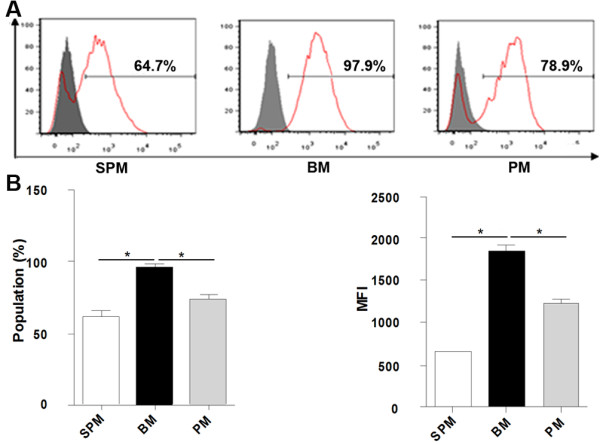
**FITC-dextran uptake assay of macrophages from the three different sources. (A)** Purified macrophages were incubated with FITC-dextran at 37°C for 45 min, and then washed extensively to remove excess FITC-dextran, followed by FACS analysis. Representative histograms are shown. Solid grey histograms represent control groups; solid red lines represent the percentage of phagocytic macrophages. **(B) **Group histograms showing both population and median fluorescence intensity (MFI) values. Data are the mean ± SEM from five separate experiments. *p < 0.05.

Phagocytic capacity of these three populations of macrophages was examined. A substantial amount of FITC-dextran was taken up by the macrophages derived from the three different sources. BMs (97.9 ± 1.2% of cells) exhibited the highest phagocytotic ability compared to SPMs (64.7 ± 3.1%) and PMs (78.9 ± 2.6%) (Figure 4A). The mean fluorescence intensity (MFI) of BMs, SPMs and PMs was 1980 ± 145, 645 ± 29 and 1232 ± 77 respectively (Figure 4B), indicating the higher phagocytotic ability of individual BMs. The MFI value of PMs was higher than SPMs indicating the higher phagocytotic capability of PMs.

### Antigen presenting capacity

**Figure 5:**
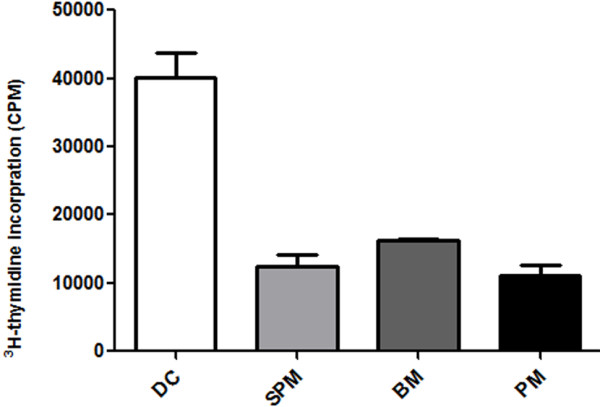
**Stimulation of CD4+ T cells by macrophages presenting OVA in [3H]-thymidine incorporation assays. **Isolated macrophages and dendritic cells (DCs) were loaded with OVA (10 μg/ml) and irradiated; then co-cultured with DO11.10 CD4+ T cells for 48 hours. Cultures were then pulsed with [3H]-thymidine, and incorporated counts determined. DCs were used as positive control. Data are the mean ± SEM from three separate experiments.

SPMs, BMs and PMs were analyzed for their ability to present OVA antigen to OVA-specific DO11.10 CD4+ T cells by [3H]-thymidine incorporation assay. DCs generated from bone marrow were used as positive control. Each of these types of macrophage exhibited a much lower OVA-specific antigen presenting ability than DCs, and there was no significant difference in the ability of presenting OVA-specific antigen among the three types of macrophage (Figure 5).

### Cytokine expression profile of SPMs, BMs and PMs

**Figure 6:**
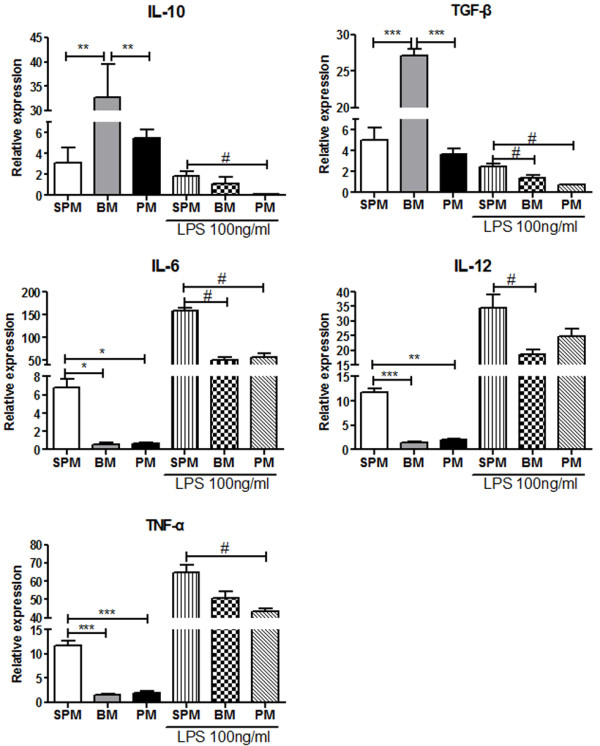
**Cytokine mRNA expression profiles of the three populations (SPMs, BMs and PMs) with and without activation with LPS. **mRNA levels of IL-10, TGF-β, IL-6, IL-12 and TNF-α in SPMs, BMs and PMs were measured by real time PCR with β-actin as the housekeeping gene; (n = 5). Values are expressed as 10x (gene of interest vs β-actin). *p < 0.05, **p < 0.01, ***p < 0.001.

Cytokine mRNA expression profiles were examined. Under resting conditions, BMs produced significantly higher levels of IL-10 and TGF-β than SPMs and PMs. SPMs produced significantly higher levels of IL-6, IL-12 and TNF-α than BMs and PMs. However, following LPS activation, SPMs still expressed high levels of pro-inflammatory cytokines (IL-6, IL-12 and TNF-α) in comparison to BMs or PMs. SPMs expressed significantly higher level of suppressive cytokine IL-10 and TGF-β than PMs. SPMs also expressed significantly higher level of TGF-β than BMs (Figure 6).

## Discussion

Macrophages have heterogeneous phenotypes and complex functions within both innate and adaptive immune responses [19]. To date, most experimental studies have been performed on BMs, isolated SPMs and PMs [1]. However, differences among macrophages from these particular sources remain unclear. In this study, the features of macrophages from spleen, bone marrow and peritoneal cavity were compared. We found that PMs appear to be more mature than SPMs and BMs, based on their morphology and surface molecular characterizatics. BMs showed the strongest capacity in both proliferation and phagocytosis among the three populations of macrophage; under resting conditions, SPMs maintained high level pro-inflammatory cytokine expression (IL-6, IL-12 and TNF-α), whereas, BMs had high level expression of suppressive cytokines (IL-10 and TGF-β); after LPS activation, SPMs expressed relatively high levels of all those cytokines.

In macrophage studies, macrophage cell lines including J774A.1, RAW264.7, P388D1 and U937 [20,21] can be used, however, continuous subculture of these cell lines may cause gene loss and impair macrophage immune functions. Therefore, macrophages from bone marrow, spleen and peritoneum in primary culture are more commonly used. To date, macrophage studies have been performed and validated extensively using BMs [22-24], but less so with SPMs and PMs. Unlike macrophages obtained directly from spleen and peritoneum, BMs can be fully differentiated *in vitro* from macrophage dendritic cell precursors [25]. Although there are many advantages in using BMs in immunological studies, such as their high yield, homogeneity and long lifespan [23], the features of BM macrophages are not fully characterized. Morphological changes of macrophages from three sources were examined to compare their maturation. Consistent with the previous studies [26], there are some similarities among SPMs, BMs and PMs with regard to their sphere and deeply stained nuclei, but SPMs and PMs contained much more cytoplasm than BMs, suggesting that BMs may be less mature then SPMs and PMs. When comparing cytoplasm of SPMs with PMs, PMs exhibited a larger size and lysosomal content than SPMs, suggesting that PMs may be more mature than SPMs. In addition to morphological analysis, surface molecular expression could also be used, at least in part, to indicate the maturity of the three populations. A study from Alatery showed that both SPMs and BMs were not fully mature and needed to undergo a further maturation *in vitro* in culture [26]. Our study detected surface molecular expression that related to macrophage maturation and function. PMs had high level MHC II and CD86 expression, whereas BMs had high level CD115 and GR-1 expression. MHC II and CD86 are expressed highly on fully functional macrophages, which also indicates their maturity [27,28]. CD115 and Gr1 are usually expressed on precursors of monocytes and macrophages, indicating that the cells are less differentiated and more immature [29]. Therefore, our study showed that PMs appear to be the most mature macrophage, followed by SPMs, then BMs. These differences are likely important considerations in the experimental use of macrophages from different sources.

Following great technical improvements in the *in vitro* generation of macrophages, they are now considered as candidates for cell therapy [17,30-32]. Currently, there is a much variation in the preparation of macrophages from different sources for therapeutic use. A recent study of muscle regeneration demonstrated the therapeutic potential of macrophages derived from bone marrow [33]. However, both the experimental and clinical use of regulatory macrophages (M2) for treating central nervous system injury relied on generation of macrophages from peripheral blood. Previously we have demonstrated the therapeutic efficacy of M2 macrophages derived from spleen, but not bone marrow, to resolve inflammation and repair the kidney injury [34-37]. We have shown a similar efficacy of M2 macrophages derived from peritoneum as from spleen (unpublished data). This demonstrates the importance of the origin of macrophages used for treating disease. In this present study, the proliferative, phagocytotic and antigen presenting ability of BMs, SPMs, and PMs were assessed. It was found that BMs exhibited the strongest proliferative capability among the three populations, with SPMs demonstrating slight and PMs no proliferative capability, suggesting that macrophages derived from spleen and peritoneum might be more functionally and phenotypically stable. This observation is consistent with our previous report that M2 macrophage generated from bone marrow rather than spleen showed strong proliferation *in vivo* and failed to protect against renal disease, apparently due to the loss of function and phenotype of macrophages linked to their proliferation ability [35]. In addition to proliferative ability, phagocytotic capacity of macrophages was assessed. BMs have been shown to maintain the highest capability of phagocytosis [38,39], which was confirmed in our study and may be an important consideration in regards to their therapeutic efficacy.

T-cell activation and proliferation is associated with many chronic inflammatory diseases, including chronic kidney disease, rheumatoid arthritis and atherosclerosis [17,40,41]. Inhibition of T-cell activation is important in effectively suppressing inflammatory responses. A previous study showed that B7-H1 binding to its receptor, PD-1, results in inhibition of antigen-induced T-cell activation [42]. High expression of B7-H1 on PMs suggests PMs might inhibit T cell activation more effectively than SPMs or BMs. Such a property of PMs indicates a greater potential for treating chronic inflammatory diseases.

Although SPMs, BMs and PMs exhibited different levels of expression of molecules involved in antigen presentation, such as MHCII, CD80 and CD86, they showed similar antigen presenting ability. Many PMs are recruited into peritoneal cavity in response to bacterial infection, in greater amount than other cell types [43,44]. In spleen, several subpopulations of macrophage have been characterized *in vivo*, including F4/80+ red pulp macrophages, MOMA-1+ marginal metallophilic macrophages, ER-TR9+ marginal zone macrophages and MOMA-2+ white pulp macrophages in mice [7]. F4/80 is prodominantly expressed on red pulp macrophages, but not on others such as marginal metallophilic macrophages, marginal zone macrophages and white pulp macrophages. Therefore, F4/80 stained cells might be less diverse and could be considered as a relative uniform population. However, other subpopulations of splenic macrophages require further study.

Comparison of cytokine expression profile of SPMs, BMs and PMs might contribute to the understanding of their distinct properties and provide a valuable reference for further macrophage related studies. The significantly higher expression of TGF-β and IL-10 by resting BMs in comparison to SPMs and PMs suggests that *in vitro* generated BMs might be potentially more likely to have a M2 phenotype. M-CSF has been shown to induce differentiation of BMs from bone marrow progenitors [45] and also to induce human macrophages into a M2 phenotype [46]. Compared to an only 1 day *in vitro* incubation time of SPMs and PMs, the requirement of 7 days stimulation of bone marrow cells with M-CSF may push them towards M2 differentiation. Combined with high proliferation and phagocytosis ability of BM, thus suggests that BMs might be less mature and phenotypically stable than SPMs and PMs, giving caution to the use of BMs in cell therapy. Alternatively, pro-inflammatory cytokines including IL-6, IL-12 and TNF-α were significantly more highly expressed on SPMs with and without activation than BMs or PMs, which may be relevant to the specific microenvironment of spleen. In spleen, SPMs play an important role in removal of red cells, which may require SPMs to produce abundant cytotoxicity-associated cytokines such as IL-12, TNF-α and IL-6 [47,48]. Therefore, cytokine expression of BMs, SPMs and PMs reflect their biological function.

## Conclusions

In summary, we report a side-by-side comparison study of macrophages derived from spleen, bone marrow and peritoneum. This study demonstrates their distinct characteristics which are likely relevant to their respective roles in immune response. It also provides a powerful reference for choosing macrophages of specific origins not only for experimental study but also for therapeutic use.

## Methods

### Animals

Six- to eight-week-old male BALB/c mice purchased from the Animal Resources Centre (Perth, Australia) were used in this study. DO11.10 mice were obtained from Animal House of Westmead Hospital (Animal Care Facility, Westmead Hospital, NSW, Australia). All animal experiments were approved by the Animal Ethics Committee of the Sydney West Area Health Service. All mice were housed in a specific pathogen-free environment and were maintained under constant temperature (22°C) and humidity, on a 12-hour light/dark cycle in the Animal House of Westmead Hospital. Then, mice were fed with acidified water and commercial mouse chow (protein 18.9%; Glen Forrest Stockfeeders, Glen Forrest, WA, Australia) *ad libitum*. Mice were sacrificed by CO_2_ inhalation.

### Preparation of SPMs, BMs, PMs, DCs and CD4+ T cells

Mice were sacrificed by CO_2_ inhalation. Spleens were dissected from abdominal cavity and filtered through a 40-μm nylon strainer. Red cell lysis buffer was used to remove red cells. A single splenic cell suspension then was obtained. FACS sorting was performed to obtain F4/80 positive and CD11c negative cells; then the harvested cells (0.5-1x10^6^) were cultured for 24 hours with complete RPMI1640 supplemented with 10% FBS, 2 mM L-glutamine, 50 U/ml penicillin, 50 μg/ml streptomycin, 10 mM HEPES (N-2-hydroxyethylpiperazine-N’-2-ethanesulfoinc acid) and 0.1 mM nonessential amino acids (all from Life Technologies) and 10 ng/ml M-CSF (R&D Systems) in 6-well plates (BD Bioscience), at 37°C. For macrophage activation, cells were stimulated with 100 ng/ml LPS (Sigma) for 24 hours. The cells were harvested by trypsin (0.5%) (Invitrogen).

Pelvic and femoral bones were dissected; and all the remaining tissue on the bones was removed. Each bone end was cut off, and bone marrow was expelled. Cells from bone marrow were cultured for 7 days with 10 ng/ml M-CSF; medium was changed every two days. Adherent cells were detached by trypsin (0.5%) digestion. FACS sorting (BD Bioscience) was performed to obtain F4/80 positive and CD11c negative cells; then the harvested cells (0.5-1×10^6^) were cultured for 24 hours in complete RPMI1640 with 10% FBS in 6-well plates, at 37°C. For macrophage activation, cells were stimulated with 100ng/ml LPS (Sigma) for 24 hours. The cells were harvested by trypsin (0.5%).

Peritoneal membrane was separated from under the abdominal musculature. 5-7 ml ice cold PBS was injected into peritoneal cavity; peritoneum was gently and completely massaged; PBS was then aspirated from peritoneal cavity. Peritoneal cells were enriched by centrifugation, then purified by FACS sorting by selecting F4/80 positive and CD11c negative population, then the harvested cells (0.5-1×10^6^) were cultured for 24 hours in complete RPMI1640 with 10% FBS in 6-well plates, at 37°C. For macrophage activation, cells were stimulated with 100ng/ml LPS (Sigma) for 24 hours. The cells were harvested by trypsin (0.5%). The purity of detached cells was assessed by Flow analysis (Additional file 1).

To obtain dendritic cells, cells from bone marrow were cultured for 7 days with 10 ng/ml GM-CSF and 10 ng/ml IL-4; medium was changed every two days. Floating cells were removed by PBS washing, adherent cells were considered as DCs.

OVA-specific CD4+ T cells were isolated from DO11.10 mice. DO11.10 mice were sacrificed by CO_2_ inhalation. Spleen were dissected from abdominal cavity and filtered through a 40-μm nylon strainer. Red cell lysis buffer was used to remove red cells. A single splenic cell suspension then was obtained and incubated with mouse CD4 MicroBeads (Miltenyi Biotec) for 15 min on ice. MACS-bead separation was performed to obtain CD4+ T cells.

### Gimesa-Wright staining and lysosome staining

Cells were cultured in 6-well plates and fixed by 100% methanol for 10 min at -20°C; and after air-drying, 1 ml of Gimesa-Wright dye (Sigma) was added into each well for 3 min at room temperature and then washed with PBS completely. Stained cells were examined under microscopy (Nikon) with magnification x400. For lysosome staining, cells were fixed by 100% methanol for 10 min at -20°C. Anti-mouse lysosome associated membrane protein 1 (LAMP1) (1/400; Abcam) was used as primary antibody and Alexa Fluor® 488 (green) goat anti-rabbit IgG (1/1000; eBioscience) was used as second antibody. DAPI was used to stain the cell nuclei (blue). Images were captured by fluorescent microscope (Olympus) with magnification x400.

### Flow cytometry analysis

The macrophages were resuspended in PBS containing 2% fetal bovine serum (FBS). Non-specific Ab binding was blocked with addition of Fc block Ab, then fluorochrome-labelled Abs against macrophage surface markers were added in a concentration of 1:200; cells were stained for 20 min on ice and washed 3 times with cold PBS. Unstained samples were prepared for cell size assessment. Data were collected with Flow Cytometer LSRII and analyzed with Flow Jo software. Abs used in this study are listed in Table 1.

**Table 1:** Antibodies for flow cytometry analysis

**Antibody**	**Flurochrome**
anti-mouse F4/80	PE-conjugated
anti-mouse CD11c	APC-conjugated
anti-mouse CD80	PE-conjugated
anti-mouse CD86	PE-conjugated
anti-mouse CD115	PE-conjugated
anti-mouse CD206	APC-conjugated
anti-mouse Gr-1	PECy7-conjugated
anti-mouse MHC II	PE-conjugated
anti-mouse B7-H1	PE-conjugated
anti-mouse B7-H2	PE-conjugated
anti-mouse B7-H3	PE-conjugated
anti-mouse B7-H4	PE-conjugated

All antibodies were from eBioscience.

### Proliferation assay

Macrophages derived from spleen, bone marrow and peritoneal cavity were purified. Then, purified macrophages were cultured in separate 6-well plates at the concentration of 1 × 10^4^ cells per well. Medium used was complete RPMI 1640 with M-CSF in two concentrations (10 ng/ml and 2 ng/ml). Medium was changed every two days. The cell number was measured by counting under microscope, on days 4, 7 and 14.

### FITC-dextran uptake assay

In order to measure macrophage phagocytic ability, the FITC-dextran uptake assay was set up by incubating cells with FITC-dextran in triplicate plates. Briefly, purified macrophages were cultured on 12-well plates at a concentration of 0.5 × 10^5^ cells/well. FITC-dextran was added into each well at a final concentration of 0.5 mg/ml, and the culture plates was incubated at 4°C and 37°C for 45min. After incubation, wells were washed extensively to remove excess FITC-dextran. Macrophages were detached by digestion with 5% trypsin. FACS analysis was performed; median fluorescence intensity (MFI) was calculated.

### [3H] thymidine incorporation assay

For analysis of *in vitro* T cell proliferation, isolated macrophages and dendritic cells (DCs) were incubated with 10 μg/ml ovalbumin (OVA) peptide 323-339 (Genscript, USA) for 60 min at 37°C. OVA-loaded cells were washed 3 times with RPMI 1640. A total number of 50,000 naive CD4+ T cells were cultured in 96-well plates with 50,000 OVA-loaded macrophages or DCs for 48 hours; then 3H-Thymidine (1 μCr/well) was added and the incubation continued for a further 16 hours. Cells were harvested using a Packard Filtermate Harvester 96 and counted by Microbeta counter (PerkinElmer, Beaconsfield, UK).

### Real time PCR analysis

RNA was extracted using the Qiagen (MD, USA) RNeasy mini kit according to the manufacturer’s instructions; For reverse transcription, first strand cDNA was transcribed from total RNA using a First Strand cDNA Synthesis Kit ((Fermantas, Australia) by following the manufacturer’s instructions. Then the SBYR Green qPCR Detection System (Invitrogen) was employed for real-time PCR. Real-time PCR amplification was carried out in Corbett Rotorgene 6000 real-time Thermo cycler using a PCR mixture containing primers, cDNA and SYBR green mastermix. Levels of mRNA expression were normalized to housekeeping gene β-actin mRNA levels. GraphPad Prism 5.0 was used for statistical analysis. The primers used in this study are listed on Table 2.

**Table 2:** Primers for real time PCR

**Gene**	**Sequences of primers**	
β-actin	Left:5^′^-GATTACTGCTCTGGCTCCTAG-3’	Right:5^′^-GCCACCGATCCACACAGAGT-3’
IL-10	Left:5^′^-CCAGTACAGCCGGGAGACA-3’	Right:5^′^-CAGCTGGTCCTTTGTTTGAAAG-3’
TGF-β	Left:5^′^-TTAGGAAGGACCTGGGTTGG-3’	Right:5^′^-AGGGCAAGGACCTTGCTGTA-3’
IL-6	Left:5^′^-CACAAGTCCGGAGAGGAGAC-3’	Right:5^′^-TTGCCATTGCACAACTCTTT-3’
IL-12	Left:5^′^-GACATCACACGGGACCAAAC-3’	Right:5^′^-TACCAAGGCACAGGGTCATC-3’
TNF-α	Left:5^′^-TGCCTATGTCTCAGCCTCTTC-3'	Right: 5^′^-GAGGCCATTTGGGAACTTCT-3'

### Statistical methods

The Student’s *T*-test was used for 2-group comparisons, and ANOVA was used for comparisons involving 3 or more groups. A P value of less than 0.05 was considered statistically significant. Values are expressed as means ± standard error (SEM).

## Authors’ contributions

CW and XY performed all the experiments under the supervision of D C.H. H and YW. QC and all other authors contributed to the experimental design. CW wrote the manuscript; D C.H. H and YW revised the manuscript. All authors approved the manuscript.

## Supplementary Material

Additional file 1 **SPMs, BMs and PMs were generated respectively, and then stained with anti-F4/80 and anti-CD11c, the gating of F4/80+ and CD11c-cells was based their isotype controls. **Data are representive of 5 separate experiments. Click here for file
