# Supplementary figures and images for: Characterization of murine macrophages from bone marrow, spleen and peritoneum
Source: BMC Immunol. 2013 Feb 5;14:6. doi: 10.1186/1471-2172-14-6 (PMC3574850; doi:10.1186/1471-2172-14-6)

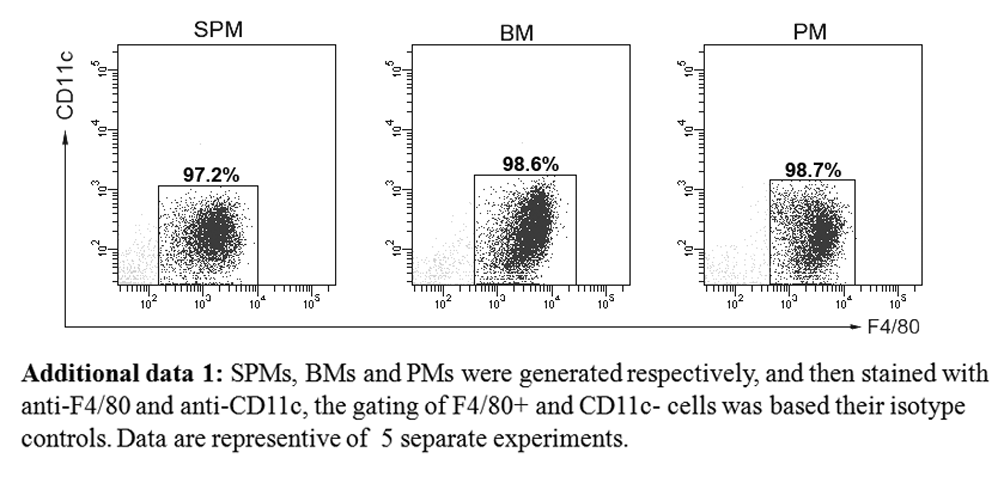

Supplement: Additional file 1 — SPMs, BMs and PMs were generated respectively, and then stained with anti-F4/80 and anti-CD11c, the gating of F4/80+ and CD11c-cells was based their isotype controls. Data are representive of 5 separate experiments. [file 1471-2172-14-6-S1.tiff]
